# Sequence embedding for fast construction of guide trees for multiple sequence alignment

**DOI:** 10.1186/1748-7188-5-21

**Published:** 2010-05-14

**Authors:** Gordon Blackshields, Fabian Sievers, Weifeng Shi, Andreas Wilm, Desmond G Higgins

**Affiliations:** 1 UCD Conway Institute of Biomolecular and Biomedical Sciences, University College Dublin, Dublin 4, Ireland

## Abstract

**Background:**

The most widely used multiple sequence alignment methods require sequences to be clustered as an initial step. Most sequence clustering methods require a full distance matrix to be computed between all pairs of sequences. This requires memory and time proportional to *N*^2 ^for *N *sequences. When *N *grows larger than 10,000 or so, this becomes increasingly prohibitive and can form a significant barrier to carrying out very large multiple alignments.

**Results:**

In this paper, we have tested variations on a class of embedding methods that have been designed for clustering large numbers of complex objects where the individual distance calculations are expensive. These methods involve embedding the sequences in a space where the similarities within a set of sequences can be closely approximated without having to compute all pair-wise distances.

**Conclusions:**

We show how this approach greatly reduces computation time and memory requirements for clustering large numbers of sequences and demonstrate the quality of the clusterings by benchmarking them as guide trees for multiple alignment. Source code is available for download from http://www.clustal.org/mbed.tgz.

## Introduction

The majority of multiple sequence alignment (MSA) methods use some form of progressive alignment [1-7]. In progressive alignment the usual first step is to compute a pair-wise distance matrix which is then used to make a so called guide tree, in order to determine the order of alignment of the input sequences. The computation of the distance matrix requires *N *(*N *- 1)/2 pair-wise comparisons, *N *being the number of sequences. Construction of the guide tree, usually has an additional time complexity of (*N*^2^) to (*N*^3^), depending on the algorithm used and its implementation. The complexity of these steps can become prohibitive when *N *becomes very large e.g. when *N *is in the tens of thousands. There are very few multiple alignment programs that can handle datasets of this size, with MUSCLE and MAFFT being the most familiar [6,7]. Some of the most accurate multiple sequence alignment methods can only routinely handle sequences numbering in the hundreds [4,8,9]. The explosive growth in the number of sequences coming from genomic studies means that the ability to cluster and align greater numbers of sequences is becoming even more important. For example, the Ribosomal Database Project [10] Release 10 consists of more than a million sequences.

In order to make very large guide trees, the first issue is the sheer number of distance calculations. For example, with 100,000 sequences, we need to compute approximately 5 billion distances to construct a complete distance matrix as needed by standard implementations of Neighbor-Joining [11] or UPGMA [12]. Even if the sequences are short, and pair-wise distance calculations can be done relatively quickly, say at a rate of 5000*s*^-1^, this still requires of the order of 1 million seconds (11.57 days) of CPU time. Just to store the distance matrix is then difficult as it will take up of the order of 20 GB of disk space and/or memory.

There are some shortcuts that can be taken to reduce the number of distance calculations needed for clustering. For example, a recent paper by Katoh and Toh [13] introduced the PartTree heuristic, which could rapidly build a very rough guide tree from an initial small number of seed sequences, using a very fast 6-mer pair-wise distance function and a divisive clustering algorithm with an average time complexity of (*N *log *N*). This algorithm was incorporated into the MAFFT suite of multiple sequence alignment programs [14]. They reported that this heuristic allowed the rapid clustering and alignment of approximately 60,000 sequences in only a few minutes. When used for a progressive alignment this considerable enhancement in speed came at a cost of several percent in alignment accuracy, as benchmarked on the Pfam database of aligned protein families [15].

In this study, we look at data embedding methods [16,17] for rapidly calculating guide trees. Our goal is to associate the sequences with a set of vectors in some *t*-dimensional *embedding space*. Embedding is done in such a way that the positioning of the vectors in the space reflects the relationships between the original sequences as best as possible. Having embedded a set of sequences, the distances between the vectors will be much faster and cheaper to calculate than distances computed using typical sequence alignment methods which require (*L*^2^) to (*L*^3^) time, *L *being the sequence length [18].

Several methods for embedding biological sequences have already been applied to protein sequences. For example, the Linial-London-Rabinovich (LLR) algorithm [16] takes a number of subsets of sequences randomly from the input dataset. Each individual sequence in the dataset is then associated with a vector whose elements are the distances between that sequence and the reference subsets (here, 'distance' is defined to be the minimum distance between sequence and subset). The number and size of the reference subsets only depends on *N*, the number of sequences, such that each embedded vector will be of dimensionality *t *= (log_2 _*N*)^2^. This algorithm was reported to offer close distance preservation in the embedded space, and was successfully applied to 38,000 sequences from the Swiss-Prot database [19], revealing many natural biological groupings. However, the original implementation meant that (*N*^2^) pair-wise distances had to be computed. SparseMap [17] was proposed as a heuristic LLR variant which was applied in much the same way as the original, but contains some heuristics to speed up the embedding process, reducing the number of pair-wise distances that had to be computed from (*N*^2^) to (*Nt*).

The reference groups in both LLR and SparseMap are generated randomly, meaning that a different embedding is found after each run. For testing purposes, this means the average result from several runs should therefore be considered when comparing methods. When applying UPGMA to the outputs from SparseMap embeddings and using these clusterings as guide trees for multiple alignments we found (results not shown) considerable differences between runs, and these differences increase as more divergent sequences are included. For these reasons we introduced SeedMap [20] which is a simplification of SparseMap which uses the same reference sequences in every run and some heuristics to make further increases in speed. SeedMap was found to be capable of producing very fast embeddings of datasets numbering in the 10s of thousands of sequences.

In this paper we look at the use of variations on SeedMap specifically for making guide trees for multiple alignment. We name the resulting method mBed and make it available with routines for sequence input and options for the output of embedded vectors or guide trees. This area of application requires high speed and moderate memory requirements for routine use by biologists. Thus, we have tried to find a method that is as simple and fast as possible while losing as little accuracy as possible compared to the use of a full distance matrix. We test accuracy using standard multiple alignment benchmarking methods [21,22]. We demonstrate the accuracy of mBed guide trees by comparing these to randomised guide trees and to guide trees directly calculated by ClustalW [5]. We also compared the accuracy of the guide trees to those from MAFFT and PartTree [7,13]. We demonstrate the scalability of the method by applying it to a set of 380,000 tRNA sequences. Finally, we show a useful by-product of the embedding process where we can easily generate ordinations of large numbers of sequences using Principal Coordinates Analysis (PCoA/PCOORD) or Multi-Dimensional Scaling (MDS) [23].

## Proposed method: mBed

Let *X *be our input dataset containing *N *sequences. We need to consider two distance metrics associated with these sequences. First we need a sequence distance [24] to establish dis-similarities between any pair of sequences *x *and *y*, denoted as *d*(*x, y*). In this paper we used the fast k-tuple distance measure of Wilbur and Lipman [25], as implemented in ClustalW [5], using the maximum possible k-tuple size of 2 (for protein), to make the distance calculation as fast as possible. Each sequence *x *will eventually be associated with a vector *F*(*x*) in some *t*-dimensional space, so we also need a metric to calculate the distance between pairs of vectors. For this we simply use the Euclidean distance metric which we denote as δ (*F*(*x*); *F*(*y*)). The embedding is considered successful then if, for all pairs of sequences, the embedded distances closely approximate the sequence distances.

In SparseMap [17] and SeedMap [20], the *t *dimensions above are distances from *t *subsets of the sequences. We refer to these subsets as *references*. In Seed Map, we aimed to improve the choice of reference groups by attempting to identify natural clusters within the dataset prior to embedding. This was found to be useful both for increasing accuracy of the embedding but also for increasing speed. In this paper, we try to gain further increases in speed by identifying single sequences from our input data *X *to act as *references*. Ideally these sequences, which shall be referred to here as "seeds", should characterise the dataset as a whole, and should therefore include representatives of natural groups/clusters within the dataset, and also include outliers.

The number of *references *chosen by the LLR method and SparseMap is a simple function of the number of sequences. In our method, however, the number of seeds chosen also depends to an extent on the nature of the data. The aim is that when the input data contains very homogeneous and similar sequences, very few seeds will be required for the embedding, and the dimensionality *t *will be small. Conversely, when more divergent sequences are considered, the number of required seeds will naturally increase. The proposed algorithm, which we name mBed, is described next.

### 1. Initial seed selection

A number of *t *sequences are initially sampled from the input dataset *X*. Following the LLR algorithm, this value is set by default to *t *= (log_2 _*N*)^2^. This sampling is referred to as *R*. Here, we chose to sample *t *sequences with constant stride from a length-sorted *X*.

The seeds that have been chosen are then compared to each other. If any two seeds are highly similar to each other (below a certain distance threshold) the shorter one is considered redundant, and is discarded. This threshold is, by default, set to zero (so that only identical sequences are excluded)

### 2. Analysis of potential seed sequences

The set of reference points *R *can now be used directly to embed the input sequences (see step 3). Alternatively, each seed sequence can be used to find extra seeds that help better characterise the dataset. This can be done in one of two ways.

'usePivotObjects' heuristic

Each seed sequence is used to find potential outliers. First, the sequence that is furthest away from the seed is identified. The sequence that is furthest away from that sequence is then returned as a new seed.

For each seed sequence *s *in *R*:

1. Let *l *be the sequence in *X *that maximises *d*(*l, s*).

2. Let *m *be the sequence in *X *that maximises *d*(*m, l*).

3. Return *m *as a new seed.

'usePivotGroups' heuristic

This works in a similar way to the *'usePivotObjects' *heuristic, but finds groups rather than single sequences. It first finds the sequence that is furthest away from the seed, and then iteratively finds the sequence that is furthest away from the group of those already chosen, i.e.:

For each seed sequence *s *in *R*:

1. Let *l *be the sequence in *X *that maximises *d*(*l, s*).

2. Let *m *be the sequence in *X *that maximises *d*(*m, s*) + *d*(*m, l*).

3. Let *n *be the sequence in *X *that maximises *d*(*n, s*) + *d*(*n, l*) + *d*(*n, m*) ...etc.

The loop terminates if the same sequence is identified more than once, or if the group reaches a set maximum size. Each member of the group is then returned as a new seed. As in step 1, before any sequences are accepted as seeds, they are first compared to those already chosen, and if they are found to be highly similar, they are rejected as seeds.

### 3. Embedding of input sequences

After the seed sequences in *R *have been chosen, all sequences in the input data are associated with a *t*-dimensional vector. This is done simply by computing the distances from all sequences to each of the *t *seeds. The distances become the coordinate values of the embedded vector, i.e. for each sequence *s *in *X*, let *F*(*s*) be the corresponding embedded vector, such that *F*(*s*) = [*d*(*s*, *R*_1_), *d*(*s*, *R*_2_)... *d*(*s*, *R*_*t*_)].

## Results

The embedding process entails the construction of vectors representing biological sequences in such a way that the distances between those vectors approximate the dis-similarities between the sequences themselves. These vector distances are orders of magnitude faster to calculate than sequence distances, and this allows us to rapidly generate a distance matrix δ (*F*(*x*), *F*(*y*)) from a set of embedded sequences. For very large numbers of sequences, perhaps numbering in the hundreds of thousands, such distance matrices can become unmanageable, due to sheer size. In these cases, the sequence vectors can be clustered using an alternative clustering method such as k-means. For this paper, our main aim is to be able to rapidly generate guide trees which can be used to make multiple alignments of the input sequences. Here, this is done by applying the UPGMA clustering algorithm to the embedded distance matrix. We then try to measure the success of the overall procedure by (i) tree comparison and (ii) comparing the multiple sequence alignments that are generated using guide trees from embedded distance matrices with those generated from full sequence distance matrices. This comparison is achieved using standard multiple alignment benchmarking procedures. Attempts at directly comparing the distance matrices using standard matrix comparison methods, such as Stress [26], proved inconclusive, and results are not shown here.

### Quality assessment by direct tree comparison

**Figure 1:**
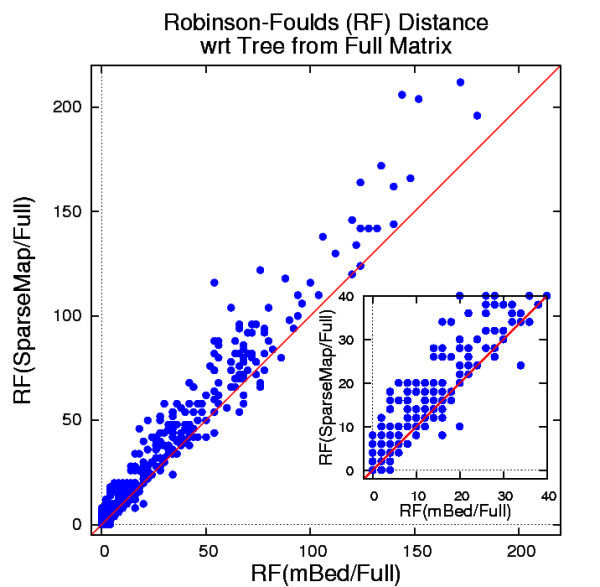
**Tree Distances**. Tree distances of SparseMap and mBed guide trees from full matrix guide trees for the BAliBase benchmark set (386 families), using the Robinson-Foulds metric. Data points above the bisectrix (red) indicate instances where the SparseMap tree is inferior to the mBed tree, and vice versa. Multiple data points may lie on top of each other.

As the mBed procedure progresses from distance matrix, via guide tree to alignment, it should prove informative to assess the quality of the intermediate step, the guide tree. For this we used the guide trees derived from (i) the full distance matrix, (ii) the SparseMap method and (iii) mBed. The full matrix guide trees were taken as the baseline. We used the Robinson-Foulds (RF) metric [27], as implemented by the treedist program of the PHYLIP suite [28], to measure the distance of the SparseMap and the mBed guide trees from the baseline. In Figure 1 we plotted the RF distance of the SparseMap guide tree from the full matrix guide tree versus the RF distance of the mBed guide tree from the full matrix guide tree for the BAliBase benchmark set of 386 test cases. As the RF measure has no immediate statistical interpretation, we simply make the qualitative observation that more points (260 out of 386) lie above the bisectrix than on it (78) or below it (48), suggesting that the SparseMap guide trees are on average 'further away' from the full matrix guide trees than the mBed guide trees.

### Initial application to multiple sequence alignment

Typically, the quality of a multiple sequence alignment is measured by comparison of the alignment to one from an independently verified reference alignment. Initially, we tested mBed on a small number of such test cases to establish the approximate speed and accuracy of mBed and its variations. The level of agreement between two alignments can be assessed using the Column Score [29], which measures the percentage of the columns of residues in the test alignment which agree with the columns in the reference alignment. We use the qscore alignment evaluation program to calculate the Column Score [6].

BAliBase [22,29] was the first large scale benchmark dataset against which alignment programs were routinely assessed. Test cases from this dataset are designed to expose new methods to many different types of alignment problems. However, the test cases are relatively small, and cannot show how alignment methods deal with very large numbers of sequences. A collection of larger test cases was therefore derived from Pfam [15,30] so that accuracy when dealing with thousands of sequences could be assessed. Each Pfam entry containing up to 10,000 sequences and which had a corresponding structural alignment for two or more of the sequences in HOMSTRAD [21] was retrieved from the database. The upper limit of 10,000 was set so that results derived from using a full distance matrix could be included for comparison.

In each test case, assessment of the overall test alignment was made by using the sequences in common between the Pfam and HOMSTRAD entry. This was usually just a relatively small number of sequences and includes those with known 3D structures. The alignment of these common sequences was then compared, using qscore. This compares the alignment generated using the guide tree, calculated using the embedded distances against the corresponding HOMSTRAD structural alignment. We show the details of the timings and qscore results for the ten largest of these test cases in Table 1. Each entry contains 9,000-10,000 protein sequences. In the same table, we also give the qscore results from using a guide tree based on a full distance matrix from sequence edit distances.

**Table 1:** mBed performance on the ten biggest Pfam/HOMSTRAD families.

Name	Size	Len	%ID	Embedding Time (s)					Distance Matrix Calculation Time (s)					Alignment Column Score (%)				
					(1)	(2)	(3)	(4)		(1)	(2)	(3)	(4)		(1)	(2)	(3)	(4)
PF01381	9993	53	23		-	25	55	136		764	57	55	175		13.3	26.7	25.3	34.7
PF00006	9796	209	43		-	134	248	280		4364	48	49	88		42.8	36.6	36.6	38.0
PF00989	9681	95	17		-	43	88	197		1281	50	51	159		46.5	33.3	31.8	34.1
PF00486	9615	75	30		-	34	69	107		950	55	52	104		63.9	92.8	64.9	89.7
PF00571	9551	119	19		-	73	143	268		1993	54	50	152		6.15	3.08	1.54	1.54
PF00097	9423	41	33		-	18	38	94		517	44	43	115		53.2	54.8	61.3	54.8
PF01479	9352	47	32		-	17	40	90		496	45	46	124		58.3	91.7	89.6	79.2
PF00046	9305	54	35		-	20	43	85		651	41	42	77		59.4	44.9	46.4	60.9
PF00550	9249	63	25		-	28	59	136		794	47	47	141		51.3	32.9	55.3	59.2
PF00149	9072	198	14		-	133	256	552		3515	47	46	172		75.4	71.9	72.3	76.1
																		
Average	9503	95	27		0	53	104	195		1533	49	48	131		47.0	48.9	48.5	52.8

The ten biggest Pfam entries containing 9,000-10,000 sequences, which have a corresponding HOMSTRAD alignment are used here. Four different methods were applied to each entry to calculate a distance matrix. These methods are: (1) the traditional process of calculating a full distance matrix from the sequence data using an alignment distance measure; (2) mBed default; (3) mBed followed by the *'usePivotObjects' *method; (4) mBed followed by the *'usePivotGroups' *method. A UPGMA guide tree is constructed from each matrix and used as a guide tree for progressive alignment of the sequences. The alignment is then scored against the corresponding HOMSTRAD structural alignment using Column Score.

(1) Full d(x, y) distance matrix; (2) mBed; (3) mBed + usePivotObjects; (4) mBed + usePivotGroups

As can be seen in Table 1, the default mBed approach (labelled (2)) requires an average of 53 seconds to embed each entry, with a further 49 seconds to generate a distance matrix from the vectors. In total, this amounts to less than 7% of the time required for computation of a full pair-wise distance matrix (1533 seconds). This saving is due to the considerable reduction in required distance evaluations, and the increased speed at which distance evaluations between the vectors can be made. The value of *t *(the number of reference or seed sequences) ranged from 143 to 169.

A UPGMA guide tree built from either distance matrix then takes an average of 5 seconds to construct (data not shown). This guide tree is passed to ClustalW to guide the alignment of the input sequences. Assessment of the alignment quality (and by association, of the embedding) is made by comparison to the corresponding HOMSTRAD entry using the Column Score (see last four columns in Table 1). On average, there is 1.9% difference in alignment quality between the mBed approach and the full distance matrix computation. There is of course a big stochastic error because we only used 10 examples, but the overall trend is clear: mBed reduces the time for guide tree computation drastically, while the alignment quality remains almost unchanged, on average.

Table 1 also shows the effect of different approaches for the selection of seeds. The variation called 'usePivotObjects' (labelled (3) in the table) brings no increase in alignment accuracy whereas 'usePivotGroups" (labelled (4)) increases the accuracy, but also almost triples the embedding time. We therefore ignore these options in the rest of this paper. The second option is of interest as it has an obvious effect on accuracy, but is not used in mBed by default. These two heuristics were just two among a long series of heuristics that were examined during the development of mBed and our earlier method, SeedMap. We include these preliminary results as it shows that there is more accuracy to be gained by careful consideration of seed/reference selection. Nonetheless, the extra computational overhead and the complicated hand optimisation that was needed to run these heuristics made us choose to drop these as default options.

### Embedding sequences scales well for large numbers of sequences

**Figure 2:**
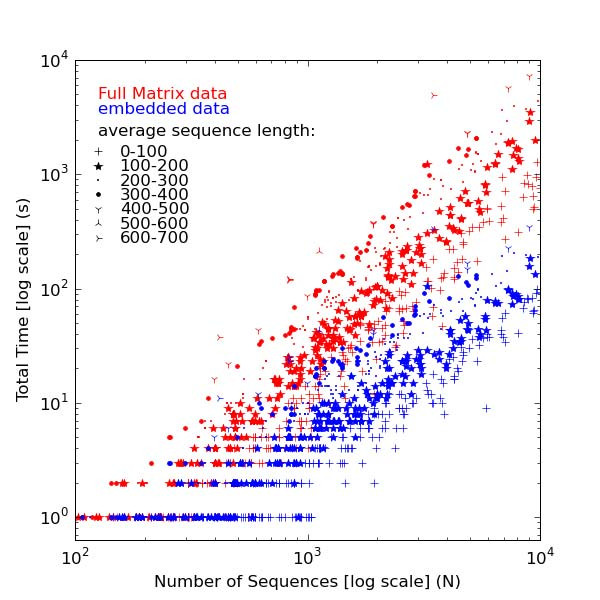
**Complexity of Embedding**. Total time required to compute a full pair-wise distance matrix (red) is plotted against time taken to embed sequences (blue) for each entry in the HOMSTRAD/Pfam dataset (containing up to 10,000 sequences per entry).

The main advantage in using a data embedding approach is the reduction in the number of pair-wise expensive distance evaluations that need to be calculated. The scatter plot in Figure 2 shows the times required to calculate a full pair-wise distance matrix directly from the sequence data (red) for each entry in the HOMSTRAD/Pfam dataset. As expected, these times scale quadratically, thus appearing linear with a slope of two on a double-log plot. However, due to the heterogeneity of the different test cases used (for example, in terms of sequence lengths), the data points do not fall neatly on to a well defined line, but within a particular region. For comparison, the total time required to (1) create a set of embedded vectors from the sequence data and (2) create a distance matrix from the vectors is plotted in blue. This plot shows a saving of an order of magnitude compared to the traditional approach, as well as a more favourable scalability (that is to say, a lesser slope).

**Figure 3:**
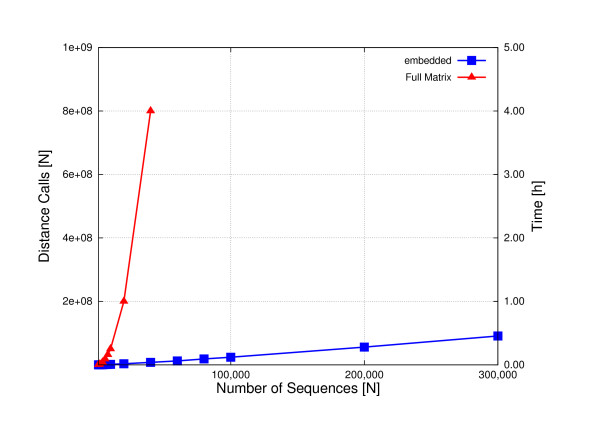
**Times for embedding up to 300,000 tRNA sequences**. Number of calls to the *d*(*x, y*) distance function made during computation of a full pair-wise distance matrix (red), plotted against number of sequences for random subsets of Rfam entry RF00005 which contains 381,602 tRNA sequences. We only show the number of calls up to 40,000 sequences. In blue we show the times for embedding subsets up to 300,000 sequences in size. The full data set takes 40 minutes to embed.

To further illustrate this scalability we use RF00005, the largest entry in the Rfam database [31]. RF00005 contains 381,601 tRNA sequences, ranging between 74-95 nucleotides in length. The similarity in length among all these sequences means that the main deciding factor in computation time, for the alignment of any subset of this dataset, is the number of sequences to be aligned. A series of subsets of different sizes were extracted from this entry and embedded. By default, the embedding process simply selects *t *sequences to act as reference points, and calculates the distances from these references to all other sequences. Essentially, this is the same as calculating *t *rows of a distance matrix. For 300,000 sequences the method selected *t *= 303 seeds. Figure 3 shows that this approach scales practically linearly with increasing values of *N*. All 381,601 tRNA sequences can be embedded in under 40 minutes, using 1 core of a 3.33 GHz Intel Xeon with 6 MB cache.

Having embedded such large numbers of sequences, it is not straightforward to use UPGMA to cluster these without taking special steps [32]. The distance matrix alone, becomes huge and difficult to generate or store in memory. Nonetheless, there are alternative, efficient clustering methods that can be used directly on the embedded vectors. For example, k-means clustering, can cluster 300,000 of these sequences, in 6 minutes (using a *k *of 300) on a single processor, after embedding.

### Choice of guide tree affects alignment quality

**Figure 4:**
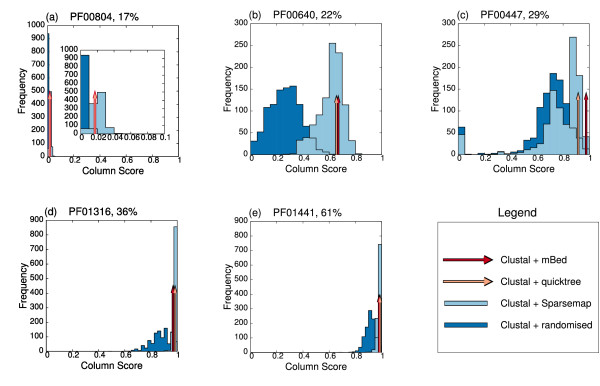
**Variation in alignment score induced by choice of guide tree**. Alignment quality scores for a collection of five test cases (a-e) taken from the HOMSTRAD/Pfam dataset, and aligned with ClustalW using guide trees generated from a variety of sources. Quality scores using guide trees from ClustalW -quicktree and from mBed are shown as arrows. Scores from 1000 randomised guide trees are shown in dark blue. Scores from 1000 SparseMap guide trees are shown in light blue.

To demonstrate the precise effects of guide tree quality on alignments of different degrees of difficulty, five test cases of 1000 sequences each, were taken from Pfam. These had between 17% and 61% pair-wise identity, on average. In each case, a guide tree was constructed using Clustal and the quality of the alignment was assessed by comparing the alignment of the included HOMSTRAD sequences against the HOMSTRAD reference alignment. Five alignments were also generated using mBed guide trees and scored. These scores are shown plotted in Figure 4.

For each test case, 1000 randomised guide trees were generated by taking the Clustal default guide tree and randomly shuffling the labels (the sequence names) on each one. This generated a distribution of scores from randomised trees of identical structure (topology and branch lengths) to the test tree. These are shown as the dark blue histograms in Figure 4. mBed is a simplification over our earlier SeedMap method [20] which is in turn related to the earlier SparseMap [17]. SparseMap, uses random seed selection and thus gives a different guide tree, each time it is run. This is an inconvenience for normal alignment purposes but in this case, it can be used to generate a range of guide trees for each of these test cases. Thus, we have also plotted the results from 1000 SparseMap runs on each part of Figure 4, using a pale blue histogram.

The first thing that can be seen is that for the most difficult of the five test cases (in panel (a) of Figure 4), it makes little difference which guide tree is used. Here, all sequences are very dissimilar and the usual beneficial effects of using a good guide tree, make little difference to the final alignment quality. This is good news and bad news. The good news is that, therefore, mBed will be no worse that using the default guide trees. The bad news is that all guide trees are ineffective anyway. For the remaining four test cases, the randomised Clustal guide trees are clearly inferior to both the default Clustal and mBed guide trees. This says that the details of the guide tree do matter a great deal, and is a very simple and direct measure of the effectiveness of progressive alignment itself. This is true, even for the easiest test cases, where the use of a good guide tree gives almost 100% correct alignments. The spread of scores from SparseMap is very noticeable in the medium difficulty test cases in panels (b) and (c). This is one reason for wanting to replace SparseMap with a method that gives the same result on every run. With very similar sequences (panels (d) and (e)), the runs are fairly uniform but with the intermediate difficulty alignments, the variation between runs is very high.

### Large-scale assessment of alignment quality

We carried out a broad assessment of alignment quality using two complete sets of test sequences. We used BAliBase because it allows comparison with other work but the numbers of test cases per reference alignment are relatively small. We therefore, also used the HOMSTRAD/Pfam test arrangement that we used earlier but now report the average accuracies across all 646 test cases.

mBed, was applied to each dataset and the results are listed in Table 2. The main mBed result is given in the last line of the table which shows results for default mBed guide trees and using ClustalW for making the alignments. Performance is also shown for alignments built using guide trees generated using our earlier SeedMap program. For comparison, at the top of the table, we give results for alignments made using default ClustalW and also with the -quicktree and -ktuple = 2 flags i.e. the mBed equivalent. We also give results for MUSCLE and MAFFT (with and without the -parttree heuristic), and also from using the PartTree output as a guide tree for ClustalW, and vice versa, using the mBed generated tree as a guide tree for MAFFT and MUSCLE.

**Table 2:** Comparison of alignment accuracy between ClustalW, MAFFT, SparseMap and mBed.

Method	Alignment Column Score (%)	
	**BAliBase**	**HOMSTRAD/Pfam**

*Guide Trees constructed internal to method*		
ClustalW	32.66	60.12
ClustalW -quicktree -ktuple=2	32.84	59.92
		
MAFFT	31.97	66.51
MAFFT -retree 1	31.24	60.09
MAFFT -retree 1 -parttree	30.04	59.27
MUSCLE	35.80	NA
MUSCLE -maxiters 1	32.04	60.45
		
*Guide Trees constructed external to method*		
MUSCLE+mBed	35.38	NA
MUSCLE -maxiters 1+ mBed	32.86	64.18
MAFFT + mBed	29.79	57.57
ClustalW + "MAFFT -retree 0 -parttree"	31.64	54.75
ClustalW + SeedMap	29.82	58.85
		
ClustalW + mBed	30.20	59.24
		

# of alignments	386	646

Average Column scores (%) are given for each method. Accuracies are measured on two datasets. The HOMSTRAD-Pfam dataset comprises 646 test cases. Each test consists of a Pfam alignment containing between 3-10,000 sequences, which has been paired with a corresponding structural alignment from HOMSTRAD.

The left hand column of results in Table 2 gives the results for the BAliBase test cases. The figures are averages across all test cases and all the numbers lie in a very narrow range with default MUSCLE performing best (35.80%), closely followed by MUSCLE using the mBed tree (35.38%). This is encouraging in that it shows that mBed does not incur any major loss in accuracy. For the HOMSTRAD/Pfam data (right hand column), we were unable to compute results for default MUSCLE due to very long running time, which is mainly caused by Muscle's iteration steps. The default version of MAFFT is the most accurate (66.51%), followed by MUSCLE with iteration switched off (60,45%). If the PartTree option is used without refinement then MAFFT's accuracy drops markedly (59.27%). On the other hand, default ClustalW starts off from a lower baseline (60.12%) but does not incur such a large drop (59.24%) if mBed is used to make the guide tree. This is the main focus of this paper. Our older SeedMap method gives slightly lower performance (58.85%).

A PartTree guide tree appears to be incompatible with the ClustalW aligner (54.75%), while an mBed tree seems to fare only slightly better as a guide tree for MAFFT (57.57%). This appears to be due to differences in how the two packages use guide trees. For example, ClustalW uses branch length information for sequence weighting. It also uses branch lengths to delay the alignment of very divergent sequences until all other sequences have been aligned. We used the -retree 0 option to generate the PartTree guide tree so as to avoid the iterative refinement step of MAFFT (Katoh, private communication). With MUSCLE, initial guide trees are generated rapidly using k-tuple counts and then refined by iteration. The initial trees are fast and simple and the alignment quality is considerable improved by the later iteration steps. We compared MUSCLE without iteration, using mBed guide trees and using the internal MUSCLE k-tuple based trees. Use of the mBed tree improves on the MUSCLE result (from 60.45% to 64.18%; iteration turned off).

### Visualisation of embedded sequences

**Figure 5:**
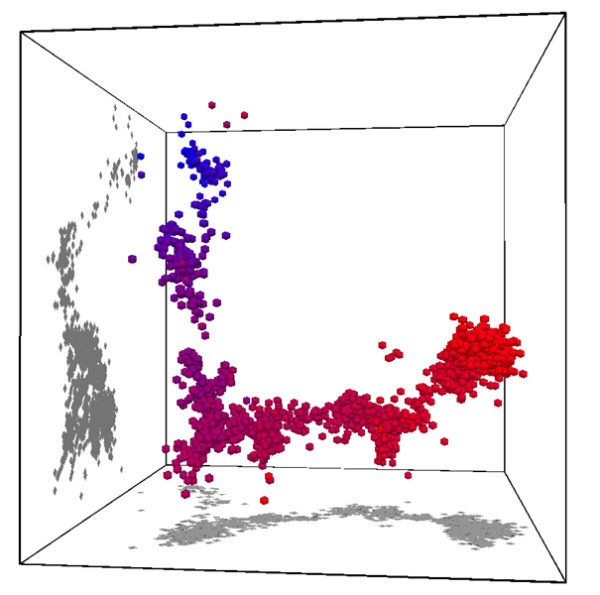
**PCA visualisation of embedded H3 Influenza virus sequences**. An embedding of 3994 GenBank haemaglutinin sequences from H3N2 influenza viruses, generated using mBed, and visualised using the first three axes of a PCA of the embedded vectors. Each sequence has been coloured by year of isolation to show the progression of sequence change between the years 1967 (blue) and 2008 (red).

Data embedding methods give the user great flexibility when visualising the relationships between sequences of interest, without the specific need to cluster or align. To give a simple example, mBed was used to generate 121 dimensional vectors for 3994 H3N2 influenza virus haemaglutinin sequences from GenBank http://www.ncbi.nlm.nih.gov/genomes/FLU, selecting 'any region' and 'any species'. These vectors were subjected to Principle Components Analysis (PCA), and the first three axes of this analysis were then used to directly visualise the virus sequences in 3D space (Figure 5). The vectors were coloured using a time-based colour scheme, representing the year of isolation for each sequence. The oldest sequences (from 1967) are coloured in blue, changing to red as time progressed (up to 2008). Such a time series is hard to visualise using simple hierarchical clustering but the almost linear progression through time is very clear using the PCA of the embedded sequences.

## Conclusions

The method that we describe here (mBed) is fast and simple but highly effective. It can be used to make guide trees of the order of 10,000 sequences using modest amounts of memory, in minutes. For very short sequences, the times can be as little as 20 seconds or so to embed the sequences. A further 5 to 10 seconds are needed to cluster the sequences using UPGMA. This is an enormous speed up over the traditional method which requires every sequence to be aligned with every sequence to generate a full distance matrix. The method also scales well and can be used to embed datasets of the size of 100s of thousands of sequences. In terms of being useful for making guide trees, the method is equivalent to the PartTree algorithm [13] which also generates guide trees, very rapidly. The two algorithms are quite different however, in detail, and mBed does have some features, for example support for branch-lengths, which make the method interesting as an alternative.

The most important criterion, ultimately, in judging an embedding of a set of sequences, is quality of the results. In earlier tests, we experimented with comparing the distance matrices from embedded sequences against full distance matrices from all-against-all comparisons using standard matrix comparison measures such as Stress [26]. The motivation was to use such comparisons to compare different seed selection methods but the results were very dataset dependant and therefore inconclusive (results not shown). As an intermediate step we compared guide trees produced by mBed and SparseMap to guide trees derived from full distance matrices. For this we used the Robinson-Foulds (RF) metric. We can see on the comparison plot that mBed guide trees are on average 'closer' to full distance matrix tree guide trees than SparseMap guide trees. In the end we chose to measure quality of the final results, using alignment benchmarking because this directly measures how well a guide tree works. This is good because it measures quality of the end product. It does not, however, say how well an embedding of a set of sequences will work for other purposes such as determining the main groups of homologous sequences in an entire database.

For our purposes, we were mainly interested in a fast way of generating guide trees for multiple alignment, especially for future versions of the ClustalW package. For this purpose, mBed works extremely well. There is a modest loss in accuracy compared to using a full distance matrix. Further, we found the guide trees worked better with ClustalW than those from PartTree although that may be due to differences between the packages and how they use guide trees. PartTree works fine when used directly with the MAFFT package.

The trees from mBed are generated strictly by grouping similar sequences rather than by attempting to accurately reconstruct phylogenetic branching orders. This would make us advise against using mBed directly for large scale phylogeny. The sequence alignments, however, may actually be improved by using guide trees that are based on similarity rather than phylogeny [6,8]. Progressive alignment works by using the guide tree to align the next most closely related sequences to each other. The most similar sequences will be the easiest to align most accurately and this delays the more difficult alignments until last. The method we have described uses a very crude method for selecting seed sequences. Ideally, we would like a much more rigorous approach that would chose seed sequences as being as representative as possible of the full diversity of sequences in a dataset. In this paper we tried a couple of modifications of the basic method and found some useful increases in accuracy but at the expense of speed. Nonetheless, the results are good, as measured by the benchmarking.

Finally, by embedding a set of sequences, we get an alternative representation of the sequences that is very flexible with regards to how the sequences can be viewed. By using the embedded sequence vectors as input to PCA, we get very elegant and clear visualisations of large numbers of sequences. For a fixed number of seed sequences one can, in principle, visualise any number of sequences, once they have been embedded. This could be used to carry out PCA on entire databases of sequences or entire outputs from high throughput sequencing runs.

## Methods

### Program Versions and Command-line Arguments

We used MAFFT version 6.705b [14], Clustal version 2.0.11 [33] and MUSCLE version 3.7 [6]. Non-default command-line arguments are given in Table 2. For evaluation of alignment quality we used qscore version 1.1 http://www.drive5.com/qscore with default arguments [6]. The Robinson-Foulds metric was computed with PHYLIP's treedist, version 3.68 http://evolution.genetics.washington.edu/phylip/general.html. The mBed source code is available on http://www.clustal.org/mbed.tgz.

### Benchmark

For benchmarking of alignment quality we used Pfam version 22.0 [15], BAliBase Version 3 [22] and HOMSTRAD, downloaded on 2009-06-09 [21]. The HOMSTRAD/Pfam benchmark comprises of Pfam entries containing up to 10,000 sequences, which had a corresponding structural alignment for two or more of the sequences in HOMSTRAD. Alignment quality was then measured on the corresponding HOMSTRAD sequences only.

## Competing interests

The authors declare that they have no competing interests.

## Authors' contributions

This project was conceived by DH and initiated and developed by GB with advice from AW and FS. The benchmarking was done by GB and FS. The software was developed by GB and FS. The influenza virus example came from WS. The final manuscript was written and approved by all authors.
